# 	Complementary treatment with oral Pidotimod plus vitamin C after laser vaporization for female genital warts: a prospective  study

**Published:** 2010-08-25

**Authors:** S Zervoudis, G Iatrakis, A Peitsidou, L Papandonopolos, MK Nikolopoulou, L Papadopoulos, R Vladareanu

**Affiliations:** * Technol. Educational Institution (TEI) of Athens, AthensGreece; ** Lito Hospital, Department of Gynecological Oncology, AthensGreece; *** Alexandra Hospital, Department of Colposcopy, AthensGreece; **** Department of Obstetrics and Gynecology, University C.Davila, BucharestRomania

**Keywords:** complementary treatment genital warts, pidotimod after laser for genital warts, pidotimod plus vitamin C for warts, immunomodulators and warts

## Abstract

This is a prospective study to assess a complementary treatment for genital warts after laser vaporization. 62 patients were enrolled in two randomized groups: A1: laser vaporization alone. A2: laser vaporization, followed with Pidotimod plus vitamin C for 2 and 1/2 months. The latter treatment shortened the time of warts remission and marginally decreased the rate of the warts' recurrence: 81% versus 67% (N.S.). Despite the non–significant difference, this complementary treatment seems to have some efficiency.

## Introduction

### Background 

For many years, remarkable interest has been shown in the immune response changes associated with different pathologic conditions, including pre–cancer lesions. It is possible that an immunodepressive condition precedes and accompanies certain diseases, although the particular condition could be transitory and reversible. Recent knowledge on this topic suggests that several compounds can modify this immune response, although it is difficult to distinguish the effect due to other contributing factors. Pidotimod (Polymod) is a synthetic compound: dipeptide, which has been shown to act as a biological response modifier (BRM). Older and recent studies indicated that pidotimod, given as monotherapy or as combined treatment, has immunostimulatory effects [1], [2]. Actually, Pidotimod showed biological and immunological activity on both the adaptive and the innate immune responses [3]. Similarly, persistent HPV infection was found lower among women reporting intake increased values of vitamin C [4]. Taking into account that Pidotimod and vitamin C, estimated separately, are considered efficient in increasing immunological activity and lowering HPV persistence respectively, our intention was to assess their combined action. 

Although there is no doubt that HPV related lesions possess a remarkable potential for spontaneous regression, if left untreated [5], immunosuppression for any reason is an established risk factor for HPV infections and related pathological conditions [6]. If we take into consideration the above–mentioned data, a faster regression of condylomatous lesions could be hypothesized after an immunostimulatory treatment. Considering that HPV lesions recur frequently after treatment, a complementary treatment should be considered to reduce the percentage of recurrences. After cryotherapy, clearance rates at three months are 63% to 92% [7]. After laser therapy, clearance rate approach is of 100% over one year [8], although recurrence can be up to 45 x0025; [7].

### Aim of the study

This is a prospective study to assess if a complementary treatment with Pidotimod (Polimod) plus vitamin C after laser vaporization for genital warts decreases the rate of recurrence.

## 	Materials and methods

Sixty–two patients (aged 17–32 years, mean age 22.3) with genital and/or perianal–perineal warts were included in the study. Mean time from first sexual intercourse and mean number of Pap smears until warts appearance was of 4.3 (years) and of 2.4 (smears) respectively. Baseline demographics of the above patients are seen in the results. The patients were recruited from a total of 65 women. Recruitment period started in June 2008 and ended in January 2009. Taking into account that genital warts usually regress postpartum and that the use of Pidotimod in the first three months of pregnancy is not recommended, pregnancy was included in the exclusion criteria. Similarly, any previous treatments were included in the exclusion criteria. Three patients were excluded from the study because they reported at least one previous therapeutic procedure in the last month (Podophylotoxine: Wartec and/or laser vaporization). 

**Figure 1:**
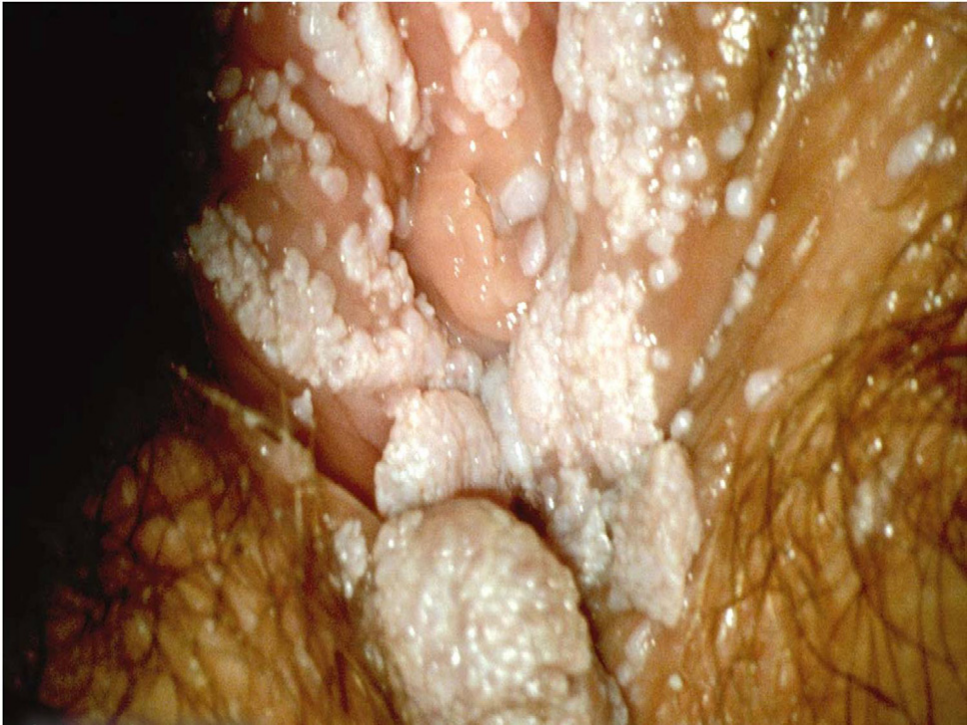
Genital warts

Patients were eligible if they had (1) perianal–perineal warts (Figure 1) diagnosed for the first time and not as a recurrence; (2) did not receive any previous treatment. As this is a preliminary study, no power calculation of the appropriate sample size was conducted and ‘all available’ patients were included in the study. 

**Figure 2:**
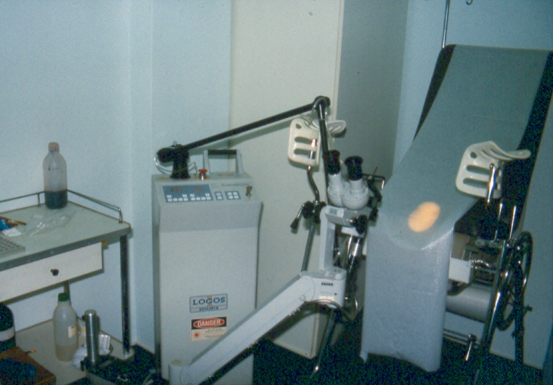
Treatment

All patients were treated with laser vaporization using CO_2_ laser with ‘painting’ motion technique (Figure 2).

Depending on the further treatment, patients were randomly subdivided in 2 groups (drawing letters A1 or A2 from a box): A1 with 30 patients and A2 with 32 patients. After the therapeutic procedure, patients belonging to the group A2 received an additional treatment with Pidotimod 800 mg (2 sachets/day) plus vitamin C (1000 mg/per day) for 15 days, and a further treatment with the same regimens in reduced doses for two more months (Pidotimod 1 sachet/day and vitamin C 500 mg/per day). Clinical and colposcopic examination was repeated at 10 weeks intervals and a final examination within 6 months was made for all the patients (a maximum of 3 examinations for each patient). Assessors were blinded to the patient's treatment allocation. 

A regulatory approval and written consents were obtained from the Scientific Hospitals' Committee and participants respectively. There was no funding for the trial. 

## Results

The mean number of sexual partners was 4.5 (range 1–9), a history of oral contraception was recorded in 6 patients and heavy smoking (more than 20 cigarettes per day) was reported by 22 patients.

Clinical and/or colposcopic disappearance of warts was diagnosed in 20 cases (67%) of group A1 and 26 cases (81%) of group A2, without clinical recurrence within six months from initial evaluation: showing a trend of increased efficacy of pidotimod and vitamin C use. Clinical disappearance of warts was obvious in 18 cases of group A1 and 25 cases of group A2 with a minute colposcopic resistance of the remaining patients. No adverse effects were reported in group A2 patients, apart for one patient who reported nausea during the second day of treatment (probably unrelated to the drug, because during the same day two more members of her family had the same symptom).

Although no statistical difference was found comparing groups A1 and A2 (p=0.2), which could be attributed to small numbers, a possible beneficial effect must not be excluded taking into account the obvious difference in percentages. In addition, shorter time of warts remission was concluded in group A2 patients as for four patients of group A2 and one patient of group A1, cure was confirmed in the second visit. Although it was not possible to determine the exact time of remission in the above cases, this ranged between 10 and 20 weeks (time between first and second visit). 

## Discussion

The cellular components of the immune system include the lymphocytes that are responsible for the production of antibodies. It seems that, in certain circumstances, a significant decrease in percentages of CD3 and CD4 populations can be observed and that pidotimod can positively impair these alterations [2]; in addition, pidotimod stimulates the release of cytokines and drives T cell proliferation [9]. Similarly, certain pathologic conditions are associated with significant changes in immune response and pidotimod is able to positively affect these disturbances. Although, our study did not include lab evaluations, a possible similar mechanism could be proposed for the marginal beneficial results of pidotimod in our patients, taking into account that CD4 reductions precede or accompany pathologic conditions of the lower genital tract. 

The efficacy of pidotimod was previously shown in vulvar papillomatosis with a good safety profile [10]. Similarly, imiquimod (Aldara [5% cream]–Graceway Pharmaceuticals), an immune response modifier used for the topical treatment of anogenital warts, has an acceptable safety profile (with local inflammatory reactions as the most frequent adverse effects).  However, complete clearance of warts showed in  < 50% of patients and recurrence rates exceeded 20% at 6 months [11]. 

Although, our results could be impaired from other co–factors (like a better ‘initial’ immunologic status of group A2), a possible beneficial effect of pidotimod on HPV related lesions could not be excluded. Furthermore, the marginally younger age, fewer sexual partners and less heavy smoking of group A2 are not significantly different from those of group A1 and thus could not impair the final findings. Further studies with an increased number of patients could clarify the possible beneficial action of pidotimod in HPV condylomatosis lesions.  

## Conclusion

Treatment with Polimod and vitamin C, after laser vaporization of genital warts could be efficient in decreasing the rate of recurrences in HPV related conditions. If this beneficial effect was confirmed in further studies, it could be attributed to the fact that the combined action of polimod and vitamin C, probably increases immunity and natural defense and decreases persistence of HPV infection. Further studies with more cases should be encouraged, in order to assess complementary systemic treatments after laser therapy or cryotherapy in genital warts, due to the high rate of recurrences.
